# Influence of SiO_2_ Embedding on the Structure, Morphology, Thermal, and Magnetic Properties of Co_0.4_Zn_0.4_Ni_0.2_Fe_2_O_4_ Particles

**DOI:** 10.3390/nano13030527

**Published:** 2023-01-28

**Authors:** Thomas Dippong, Erika Andrea Levei, Iosif Grigore Deac, Mihaela Diana Lazar, Oana Cadar

**Affiliations:** 1 Faculty of Science, Technical University of Cluj-Napoca, 76 Victoriei Street, 430122 Baia Mare, Romania; 2 INCDO-INOE 2000, Research Institute for Analytical Instrumentation, 67 Donath Street, 400293 Cluj-Napoca, Romania; 3 Faculty of Physics, Babes-Bolyai University, 1 Kogalniceanu Street, 400084 Cluj-Napoca, Romania; 4 National Institute for Research and Development of Isotopic and Molecular Technologies, 67-103 Donath Street, 400293 Cluj-Napoca, Romania

**Keywords:** Co_0.4_Zn_0.4_Ni_0.2_Fe_2_O_4_, silica matrix, crystalline phase, annealing temperature, magnetic behavior

## Abstract

(Co_0.4_Zn_0.4_Ni_0.2_Fe_2_O_4_)_α_(SiO_2_)_(100−α)_ samples obtained by embedding Co_0.4_Zn_0.4_Ni_0.2_Fe_2_O_4_ nanoparticles in SiO_2_ in various proportions were synthesized by sol-gel process and characterized using thermal analysis, Fourier-transform infrared spectroscopy, X-ray diffraction, transmission electron microscopy, inductively coupled plasma optical emission spectrometry, and magnetic measurements. Poorly crystalline Co–Zn–Ni ferrite at low annealing temperatures (500 °C) and highly crystalline Co–Zn–Ni ferrite together with traces of crystalline Fe_2_SiO_4_ (800 °C) and SiO_2_ (tridymite and cristobalite) (1200 °C) were obtained. At 1200 °C, large spherical particles with size increasing with the ferrite content (36–120 nm) were obtained. Specific surface area increased with the SiO_2_ content and decreased with the annealing temperature above 500 °C. Magnetic properties were enhanced with the increase in ferrite content and annealing temperature.

## 1. Introduction

Spinel ferrite nanoparticles are widely studied due to their outstanding electrical and magnetic properties, high thermal and chemical stability, and applicability in different areas such as electronic, microwave, and communication devices, information storage systems, ferrofluid technology, solid oxide fuel cell, gas sensors, magnetocaloric refrigeration, and medical diagnosis [1,2,3,4]. The physicochemical properties of ferrites are determined by the preparation method, heat treatment, and chemical composition, as well as the type, stoichiometric ratio, and distribution of cations [5,6,7,8,9]. The particle morphology and surface coating may also influence the magnetic behavior of ferrites having the same compositions [10].

The synthesis route is a key factor in high-purity spinel ferrite nanoparticles’ preparation [5,9,11]. The most common ways to synthesize nanostructured ferrites are sol-gel, solid-phase, hydrothermal, coprecipitation, auto combustion, sonochemical, microwave refluxing, etc. [7,10,11]. The solid-state synthesis produces nanoparticles with high yields and well-controllable grain size [3]. At the same time, the conventional ceramic method produces particles in the micrometer range that tend to agglomerate due to slow reaction kinetics [11]. Generally, wet chemical synthesis methods such as hydrothermal, sol-gel, and auto combustion are used to produce high-purity crystalline ferrite nanoparticles at low annealing temperatures [2,3,11]. The sol-gel process allows the easy, low-cost production of ferrite nanocomposites with controlled structure and properties [12]. Moreover, the sol-gel process may produce nanocomposite materials comprising highly dispersed magnetic ferrite nanoparticles [13,14,15,16]. This method consists of incorporating metal nitrates in tetraethyl orthosilicate (TEOS), polycondensation of the SiO_2_ network, thermal-assisted formation of metal-carboxylate precursors in the reaction between the metal ions and diol, and thermal decomposition of carboxylate precursors into a hybrid oxidic system [14,15,16].

In the soft ferrites family, ZnFe_2_O_4_ is a spinel, with tetrahedral (A) sites occupied by Zn^2+^ ions and octahedral (B) sites by Fe^3+^ ions; NiFe_2_O_4_ is an inverse spinel, with Ni^2+^ ions occupying the octahedral (B) sites and Fe^3+^ ions equally distributed between octahedral (B) and tetrahedral (A) sites; whereas CoFe_2_O_4_ is a mixed spinel with high inversion degree [6]. The high electrical resistivity of NiFe_2_O_4_ is due to the lack of electron hopping, as Ni exists only in the divalent form [6]. By partial substitution of Ni^2+^ with Zn^2+^ in NiFe_2_O_4_, the Zn^2+^ ions will occupy the tetrahedral (A) sites, forcing the Fe^3+^ ions to occupy both octahedral (B) and tetrahedral (A) sites. This arrangement of the cations will increase the saturation magnetization (*M_S_*) of Ni–Zn ferrite, compared to that of NiFe_2_O_4_ and ZnFe_2_O_4_ [6,10,17]. Hence, by varying the substitution degree of the divalent cation, the magnetic behavior of Ni-Zn ferrites can be enhanced, making them fit for a broad range of applications [2,6,17,18]. Besides the high *M_S_*, Ni–Zn ferrites also present large electrical resistivity, narrow dielectric loss, low coercivity, good mechanical hardness, high magnetic permeability, and high operating frequency, which make them potential candidates for transformer cores, microwave devices, noise filters, recording heads, magnetic fluids, chokes, coils, etc., [2,3,4,7].

Ni–Zn ferrite has an inverse spinel structure with Fe^3+^ ions occupying both tetrahedral (A) and octahedral (B) sites, Ni^2+^ ions preferably located in octahedral (B) sites, and Zn^2+^ ions in tetrahedral (A) sites [3]. Adding Co^2+^ to Ni–Zn ferrite induces magnetic anisotropy and reduces the permeability due to the preferential orientation of the Co^2+^ ions’ magnetic moment along a particular direction [6,7]. The low dielectric loss, low magnetic loss, high saturation magnetizations, and high resistivity of mixed Co–Zn–Ni ferrites make them widely used as capacitors, filters, magnetic antennas, and absorbing materials [5,19,20].

The SiO_2_ embedding is used to control the particle size, reduce the particle agglomeration, and enhance the ferrites’ magnetic properties and biocompatibility, as SiO_2_ is biologically inert and diminishes the inflammatory risk [12,14]. One of the most used network-forming agents in sol-gel synthesis is TEOS, as it has a short gelation time, produces strong networks with moderate reactivity, and allows the embedding of both organic and inorganic molecules [14,15,16]. Previous studies demonstrated that transitional metal ferrites embedded in the SiO_2_ matrix display high magnetocrystalline anisotropy, unique magnetic structure, and high correlation between the coercivity, crystallite sizes, and annealing temperature [14,15,16]. Also, the partial substitution of Zn^2+^ ions by Co^2+^ ions in Zn–Ni ferrite was expected to enhance the magnetic properties of the nanoparticles.

This study investigates the relationship between the Co_0.4_Zn_0.4_Ni_0.2_Fe_2_O_4_ content embedded in the SiO_2_ matrix and the crystallite size, specific surface area, porosity particle size, thermal behavior, and magnetic properties (saturation magnetization–*M_S_*, remanent magnetization–*M_R_*, coercive field–*H_c_*, magnetic anisotropy–*K*), and the Co_0.4_Zn_0.4_Ni_0.2_Fe_2_O_4_ content in the SiO_2_ matrix, at different annealing temperatures.

## 2. Materials and Methods

All chemical reagents were of analytical grade and were purchased from Merck (Darmstadt, Germany). (Co_0.4_Zn_0.4_Ni_0.2_Fe_2_O_4_)_α_(SiO_2_)_(100−α)_ samples were produced by sol-gel process dissolving Co(NO_3_)_2_∙6H_2_O, Zn(NO_3_)_2_·6H_2_O, Ni(NO_3_)_2_∙6H_2_O, and Fe(NO_3_)_3_∙9H_2_O in 1,3-propanediol (1,3PD), in a molar ratio of 0.4:0.4:0.2:2:8. Afterwards, TEOS dissolved in ethanol was added to the nitrate-1,3PD mixture, using 0:2 (α = 0%), 0.5:1.5 (α = 25%), 1:1 (α = 50%), 1.5:0.5 (α = 75%), and 2:0 (α = 100%) NO_3_:TEOS molar ratio. Diluted nitric acid was slowly added till the reaction mixture reached pH = 2 and then, the mixture was thoroughly stirred for 1 h. The obtained samples were dried at 40 °C for 5 h and 300 °C for 5 h, powdered in an agate mortar and annealed for 5 h at 500, 800, and 1200 °C, respectively, using an LT9 muffle furnace (Nabertherm, Lilienthal, Germany).

The thermal behavior of samples was studied by thermogravimetry (TG) and differential thermal analysis (DTA) by using a Q600 SDT (TA Instruments, New Castle, DE, USA) thermal analyzer, in air, up to 1200 °C, with a 10 °C/min^-^ heating rate using an SDT Q600 thermogravimeter and alumina standards. A D8 Advance (Bruker, Karlsruhe, Germany) diffractometer equipped with a LynxEye linear detector was used for the investigation of crystalline phases, using the CuKα radiation (λ = 1.54060 Å) in the 2θ range 10–80°. The FT-IR spectra of the samples were recorded using a Spectrum BX II (Perkin Elmer, Waltham, MA, USA) Fourier-transform infrared (FT-IR) spectrometer, while the composition of Ni-Zn-Co ferrites was confirmed by Perkin Elmer Optima 5300 DV (Norwalk, CT, USA) inductively coupled plasma optical emission spectrometry (ICP-OES) after aqua regia digestion using a Speedwave Xpert (Berghof, Germany) microwave digestion system. N_2_ adsorption-desorption isotherms were recorded at −196 °C by a Sorptomatic 1990 (Thermo Fisher Scientific, Waltham, MA, USA) instrument, which was used for calculation of the specific surface area (SSA) using the Brunauer–Emmett–Teller (BET) model. The particle morphology was investigated using a transmission electron microscope (TEM, HD-2700, Hitachi, Tokyo, Japan) and a digital image recording system on samples deposited on carbon-coated copper grids. The average particle size was estimated from TEM measurements using the UTHSCSA ImageTool image software for over 100 nanoparticles in each sample. The hysteresis loops were recorded in magnetic fields between −2 to 2 T, at room temperature, and magnetization versus applied field was measured on samples embedded in an epoxy matrix by a 7400 vibrating-sample magnetometer (VSM, Lake Shore Cryotronics, Westerville, OH, USA). The magnetic measurement uncertainty was 10%.

## 3. Results and Discussion

**Figure 1:**
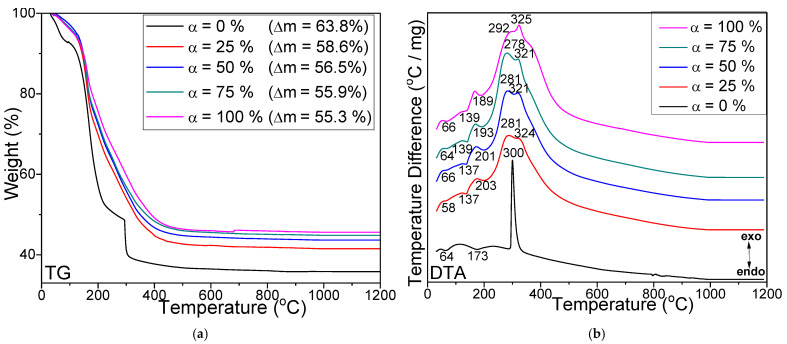
TG (**a**) and DTA (**b**) curves of (Co_0.4_Zn_0.4_Ni_0.2_Fe_2_O_4_)_α_(SiO_2_)_100−α_ samples dried at 40 °C.

The TG (Figure 1a) and DTA curves (Figure 1b indicates the maximum of the exothermic and endothermic effects, respectively) of sample α = 0% dried at 40 °C show two weak endothermic effects at 64 and 173 °C attributed to the loss of water from TEOS and an intense exothermic effect at 300 °C ascribed to 1,3PD decomposition [12]. These two processes result in a mass loss of 63.8% [12,15,16].

For samples α = 25, 50, 75, and 100%, the total mass loss slightly decreases (58.6–55.3%) with the Co_0.4_Zn_0.4_Ni_0.2_Fe_2_O_4_ content embedded in the SiO_2_ matrix. The formation of Co, Zn, and Ni malonates is indicated by the endothermic effect at 137–139 °C, whereas the formation of Fe malonate is indicated by the exothermic effect at 189–203 °C [12,14,15,16]. The exothermic effects at 278–292 °C are attributed to Co, Zn, and Ni malonates’ decomposition, while those at 321–325 °C are attributed to Fe malonates’ decomposition [12,14,15,16]. The temperature corresponding to the formation of divalent metal (Co, Ni, Zn) malonates slightly increases, whereas that of trivalent metal (Fe) malonates slightly decreases [12,14,15,16]. The transformations of the SiO_2_ matrix during the thermal process make it challenging to delimitate the effects ascribed to malonate precursors’ formation and decomposition [12,14,15,16].

**Figure 2:**
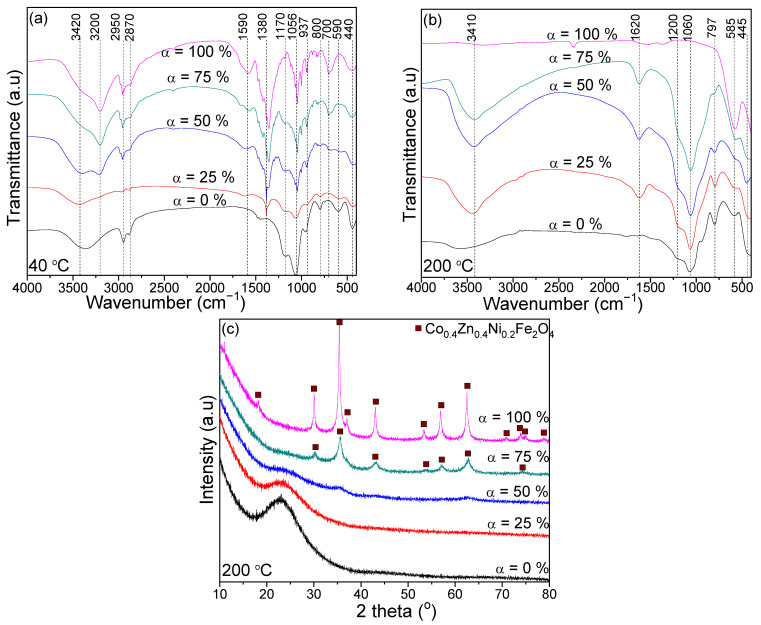
FT-IR spectra of (Co_0.4_Zn_0.4_Ni_0.2_Fe_2_O_4_)_α_ (SiO_2_)_100−α_ samples heated at 40 °C (**a**) and 300 °C (**b**) and XRD patterns of (Co_0.4_Zn_0.4_Ni_0.2_Fe_2_O_4_)_α_ (SiO_2_)_100−α_ samples at 300 °C (**c**).

Except for sample α = 0%, the FT-IR spectra of samples dried at 40 °C (Figure 2a) show a band at around 1380 cm^−1^, characteristic of nitrates. This band is missing for the samples heated at 300 °C, confirming the metal malonates’ formation and nitrates’ decomposition up to 300 °C [12,15,16]. For samples dried at 40 °C, the band at 1590–1620 cm^−1^ is specific to O–H vibrations in 1,3PD and adsorbed molecular water, and the bands at around 2950 and 2870 cm^−1^ are specific to stretching vibration of C-H in the methylene groups of 1,3PD. This band does not appear in the FT-IR spectra of samples annealed at high temperatures, indicating the precursor’s decomposition [15,16,21].

In the FT-IR spectra of sample α = 25–100% heated at 300 °C (Figure 2b), the band at around 1620 cm^−1^ characteristic to C=O of COO– groups’ vibration indicates the formation of malonate-metal complexes [15,16,21]. For samples α = 25–100%, the bands at 585–590 and 440–445 cm^−1^ indicate the presence of M-O bonds in tetrahedral (A) and octahedral (B) sites, respectively [15,16,21]. For samples α = 0–75% heated both at 40 and 300 °C, the characteristic bands for Si–O (440–445 cm^−1^) and Si–O–Si (1170–1200, 1056–1060, 792–829, 797–800, 585–590 cm^−1^) vibration suggests the formation of the SiO_2_ matrix [12,15,21]. The bands at 3420–3410 cm^−1^ are attributed to the vibration of O-H and hydrogen bonds in 1,3PD (40 °C) and metal malonates (300 °C) [15,16].

For sample α = 100% annealed at 300 °C (Figure 2c), the presence of well-defined, single-phase Co_0.4_Zn_0.4_Ni_0.2_Fe_2_O_4_ containing low-crystallized CoFe_2_O_4_ (JCPDS card no. 22–1086), ZnFe_2_O_4_ (JCPDS card no. 70–6491), and NiFe_2_O_4_ (JCPDS card no. 74–2081) is observed. The peaks pointed out at 2θ values of 18.29, 30.08, 35.43, 37.05, 43.05, 53.05, 56.97, 62.58, 70.95, 74.01, 75.01, 78.97° (CoFe_2_O_4_); 18.25, 30.01, 35.35, 36.98, 42.96, 53.30, 56.81, 62.39, 70.77, 73.79, 74.80, 78.74° (ZnFe_2_O_4_); and 18.42, 30.29, 35.68, 37.33, 43.37, 53.82, 57.37, 63.01, 71.51, 74.57, 75.58, 79.59° (NiFe_2_O_4_), corresponding to Miller indices of (111), (220), (311), (222), (400), (422), (511), (440), (620), (533), (622), and (444) confirms the formation of single-phase spinel-like structure (space group *Fd3m*). The degree of crystallinity was calculated using the highest intensity peak of spinel ferrite (311) [8,21,22]. In sample α = 75% annealed at 300 °C, the presence of Co_0.4_Zn_0.4_Ni_0.2_Fe_2_O_4_ is also observed, although the degree of crystallinity is lower than in sample α = 100%. In samples with low ferrite content α = 0, 25, and 50%, the crystalline ferrite is not remarked; the intensity of halo between 16 and 30° (2θ) ascribed to the amorphous SiO_2_ matrix increases with the SiO_2_ content.

**Figure 3:**
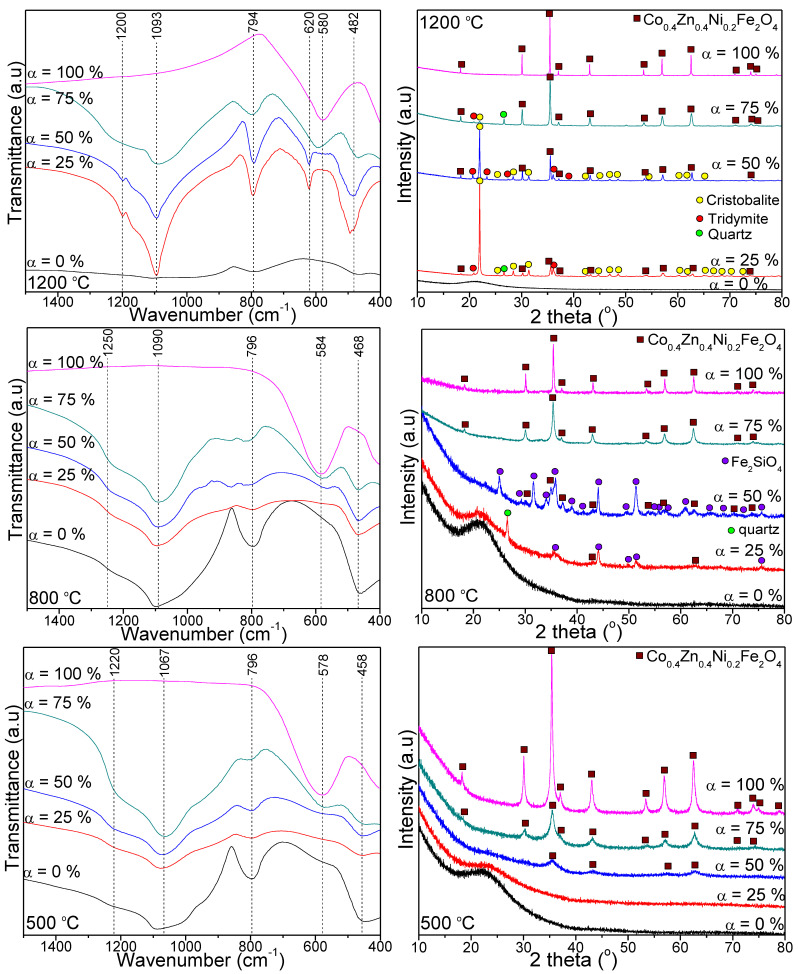
FT-IR spectra (**left**) and XRD patterns (**right**) of (Co_0.4_Zn_0.4_Ni_0.2_Fe_2_O_4_)_α_ (SiO_2_)_100−α_ samples annealed at 500, 800, and 1200 °C.

Figure 3 displays the FT-IR spectra (left) and XRD patterns (right) of samples annealed at 500, 800, and 1200 °C. For samples α = 0–75%, the occurrence of SiO_2_ matrix is supported by the symmetric and asymmetric stretching vibrations of SiO_4_ tetrahedron (794–796 cm^−1^), the Si–O–Si stretching vibrations (1067–1093 cm^−1^), the shoulder at 1220–1250 cm^−1^ and the Si–O bond vibration (458–482 cm^−1^) [12,15,16]. These bands are lacking for samples α = 100%, the bands at 578–584 cm^−1^ being ascribed to Zn–O, Co–O and Ni–O vibrations, and at around 400 cm^−1^, they are ascribed to Fe–O bonds’ vibration [12,15,16]. For samples with high SiO_2_ content (α = 25%) annealed at 1200 °C, the vibration band at 620 cm^−1^ is attributed to Si–O–Si cyclic structures [12,15,16].

The XRD patterns of samples α = 100% annealed at 500, 800, and 1200 °C (Figure 3) display no impurities or unreacted Fe, Ni, Co, and Zn oxides, the broadening of diffraction peaks being ascribed to ultrafine Co_0.4_Zn_0.4_Ni_0.2_Fe_2_O_4_ particles [12,15,16]. The intensity of diffraction peaks matching to ferrites increases at high annealing temperatures indicating a high degree of crystallinity, high crystal nucleation (owing to the small growth rate and homogenous distribution), and large crystallites (owing to the coalescence process) [6,8,14,15,16,22]. The degree of crystallinity (DC) was determined as the ratio between the area under all diffraction peaks and the total area under the amorphous halo and diffraction peaks [14,15,16]. The intensity of the main diffraction peak of cubic spinel ferrite at (311) plane was considered as a measure of the degree of crystallinity [14,15,16].

By annealing at 500 °C, the samples α = 25, 50 and 75% display single-phase Co_0.4_Zn_0.4_Ni_0.2_Fe_2_O_4_, but less crystallized than the sample α = 100%, the degree of crystallinity increases with the ferrite content embedded in the SiO_2_ matrix. With sample α = 75% annealed at 800 °C, single phase Co_0.4_Zn_0.4_Ni_0.2_Fe_2_O_4_ is observed, while for sample α = 50%, the presence of trace Fe_2_SiO_4_ (JCPDS card no. 70–1861) with peaks at 2θ values of 25.04, 29.30, 31.62, 34.97, 35.91, 37.29, 41.2, 43.67, 51.39, 54.67, 55.73, 57.42, 61.17, 65.46, 68.38, 72.11, and 75.43° associated with Miller indices of (111), (002) (130), (131), (112), (200), (220), (132), (222), (061), (133), (043), (062), (162), (322), (080), and (303) is also remarked.

The formation of Fe_2_SiO_4_ appears due to the incomplete reduction of Fe^3+^ to Fe^2+^, which further reacts with the SiO_2_ matrix, forming Fe_2_SiO_4_ [16].

For sample α = 25% annealed at 800 °C, the main phase of Co_0.4_Zn_0.4_Ni_0.2_Fe_2_O_4_ is accompanied by Fe_2_SiO_4_ and quartz (JCPDS card no. 79–1910) at 2θ value of 26.6° corresponding to Miller indices of (011), whereas for sample α = 75% annealed at 1200 °C, beside the main phase of Co_0.4_Zn_0.4_Ni_0.2_Fe_2_O_4_, crystalline phases of SiO_2_ (quartz and tridymite (JCPDS card no. 042–1401)) with peaks at 2θ values of 20.76, 23.38, 27.49, 36.17, and 37.72 corresponding to Miller indices of (220), (222), (420), (040), and (240) are remarked. Unexpectedly, the samples α = 25 and 50% annealed at 1200 °C display cristobalite (JCPDS card no. 39–1425) with peaks at 2θ values of 21.98, 25.32, 28.43, 31.46, 42.66, 44.84, 47.06, 48.61, 56.22, 60.30, 62.01, 65.65, 66.81, 68.67, 69.42, 69.79, 70.54, and 72.69 associated with the Miller indices of (101), (110), (111), (102), (321), (202), (113), (212), (104), (311), (302), (204), (223), (214), (321), (303), (105), and (313) as the main crystalline phase attended by Co_0.4_Zn_0.4_Ni_0.2_Fe_2_O_4_ and tridymite. Additionally, for sample α = 25%, quartz is also present. The intensity of diffraction peaks belonging to the cristobalite increases with the SiO_2_ content.

At all annealing temperatures, for samples with no ferrite content (α = 0%), no crystalline phases are observed and the halo between 16 and 30° (2θ) matches the amorphous SiO_2_ matrix. A possible explanation for the absence of crystalline phases corresponding to the SiO_2_ could be the difficult diffusion of oxygen within the pores of the silica matrix [14,15,16]. Usually, the amorphous phase content is proportional to the area under the diffraction halo, but not all the amorphous phases produce diffraction halos due to the lack of a significant local order. The area under the amorphous halo and the total area of the diffraction peaks were used to explore the evolution of amorphous and crystalline phases [14,15,16]. Similar behavior is observed for the sample with high SiO_2_ matrix content (α = 25%) annealed at 500 °C. Consequently, low annealing temperature and high SiO_2_ content led to highly amorphous content. However, not all the amorphous phases imply diffraction halos owing to the absence of a large local order [14,21].

The average crystallite size (D_XRD_) was calculated using the Scherrer formula (Equation (1)):

(1) $$D_{\mathrm{XRD}}=\frac{0.9\cdot \lambda }{\beta \cdot \cos\theta }$$

where λ is the wavelength of CuK_α_ radiation (1.5406 Å), β is the broadening of full width at half-maximum intensity (FWHM), and θ is the Bragg angle (°) [12,13,14,15,16].

The average crystallite size increases with the annealing temperature and Co_0.4_Zn_0.4_Ni_0.2_Fe_2_O_4_ content by the grain growth blocking effect of the SiO_2_ matrix (see Table 1) [14,15,16]. The samples with low ferrite content comprise both amorphous and crystalline phases. The changes in crystallite size may be associated with the influence of the SiO_2_ matrix on the grain growth and lattice strains, in such a way that SiO_2_ content increase, while the annealing temperature reduces the grain growth [23,24,25]. The largest crystallite size was obtained for non-embedded Co_0.4_Zn_0.4_Ni_0.2_Fe_2_O_4_ (α = 100%), following the assumption that the SiO_2_ matrix contributes to the reduction of crystallite size. One plausible explanation could be the improvement of the crystal-nuclei coalescence process, which occurs at high annealing temperatures (1200 °C). In addition, the annealing temperature reduces lattice strains and defects [14,15,16].

The lattice constant (a) was calculated using Bragg’s law with Nelson–Riley function according to Equation (2) [14,15,16]:

(2) $$a=\frac{\lambda \sqrt{h^{2}+k^{2}+l^{2}}}{2\cdot \sin\theta }$$

where λ is the wavelength of CuK_α_ radiation (1.5406 Å) [15,16].

The lattice constant (a) increases with the Co_0.4_Zn_0.4_Ni_0.2_Fe_2_O_4_ content embedded in the SiO_2_ matrix and decreases with the annealing temperature (Table 1). The high surface energy and tension, surface dipole interactions, and the cation distribution inside the nanocrystallite do not produce the lattice shrinking [14,15,16,26]. The tetrahedral (A) site (0.52 Å) displays a smaller radius than the octahedral (B) site (0.81 Å) [2], while the ionic radius of Co^2+^ (0.75), Zn^2+^ (0.74,) and Ni^2+^ (0.69 Å) are larger than the ionic radius of Fe^3+^ (0.64 Å) [15]. By increasing the number of Fe^3+^ ions in the octahedral (B) sites, the system changes from inverse spinel to normal spinel structure [14,15,16]. Consequently, adding Co^2+^, Zn^2+,^ and Ni^2+^ ions lead to a strained lattice.

The elemental composition is confirmed by the Co/Zn/Ni/Fe molar ratio using the MW/ICP-OES analysis (Table 1). The best experimental and theoretical data correlation is observed for the samples annealed at 1200 °C. For samples α = 25 and 50% annealed at 800 °C, the higher Fe content confirms the presence of Fe_2_SiO_4_ as a secondary phase observed by XRD.

**Figure 4:**
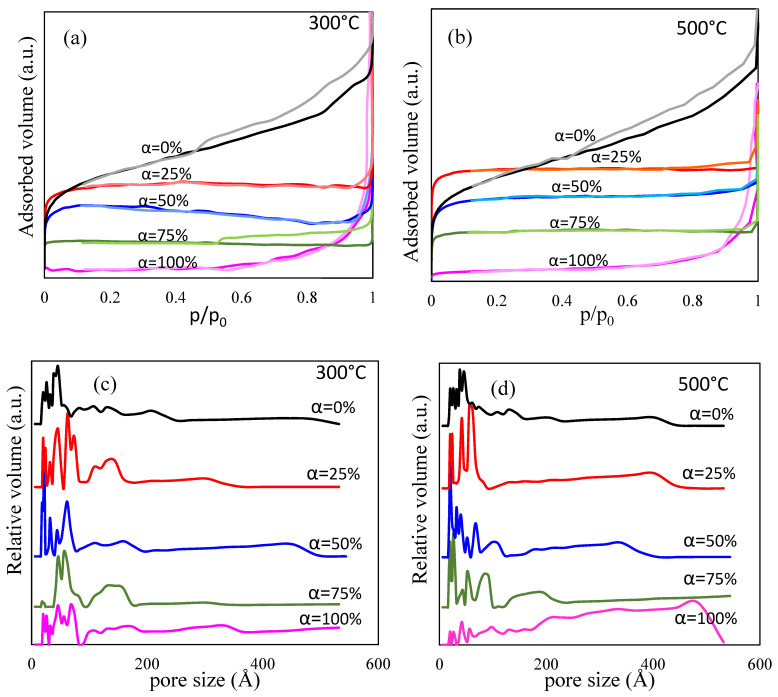
N_2_ adsorption-desorption isotherms of (Co_0.4_Zn_0.4_Ni_0.2_Fe_2_O_4_)_α_(SiO_2_)_100−α_ (α = 0, 25, 50, 75, and 100%) samples annealed at 300 °C (**a**) and 500 °C (**b**) pore size distribution at 300 °C (**c**) and 500 °C (**d**).

The shape of N_2_ adsorption-desorption isotherms of (Co_0.4_Zn_0.4_Ni_0.2_Fe_2_O_4_)_α_(SiO_2_)_100−α_ (α = 0, 25, 50, 75, and 100%) samples annealed at 300 and 500 °C (Figure 4a,b) is preserved, confirming the stability of the porous structure up to 500 °C. The isotherms of samples annealed at 800 and 1200 °C could not be recorded, indicating the breakdown of porous structure at temperatures above 500 °C. The isotherm for SiO_2_ (α = 0%) is of type IV, and for samples α = 25, 50, and 75% is of type I [27].

The SSA decreases with the increase of α, by the increase of D_XRD_ (Table 1). For SiO_2_ (α = 0%) and ferrite (α = 100%) samples, the SSA does not depend on the annealing temperature, while for samples α = 25, 50, and 75% annealed at 500 °C, an increase of SSA value was observed. A possible explanation could be the better organization and crystallization of samples annealed at 500 °C than at 300 °C. The pore size distribution (Figure 4c,d) shows that all samples contain different-sized pores up to 550 Å. The pores are generally under 100 Å in samples annealed at 500 °C and up to 200 Å in samples annealed at 300 °C, respectively. These results follow the variation of SSA described above.

**Figure 5:**
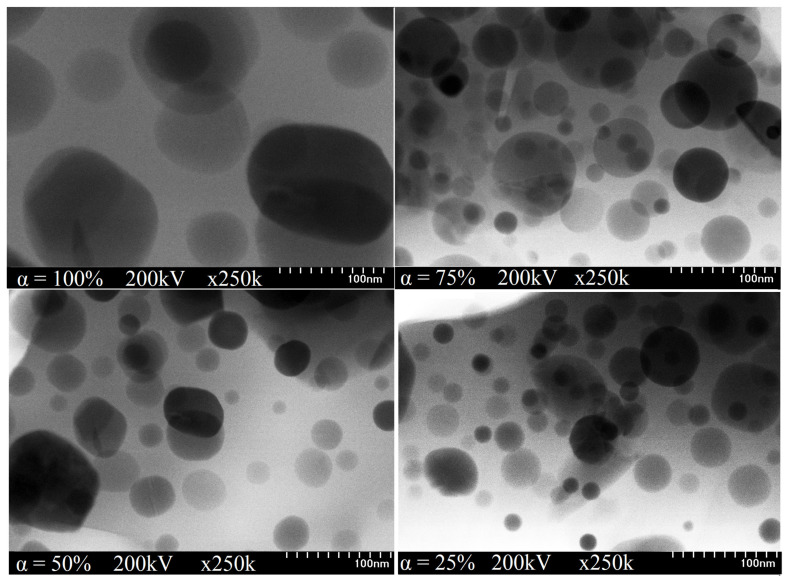
TEM images of (Co_0.4_Zn_0.4_Ni_0.2_Fe_2_O_4_)_α_(SiO_2_)_100−α_ samples (α = 25–100%) annealed at 1200 °C.

**Figure 6:**
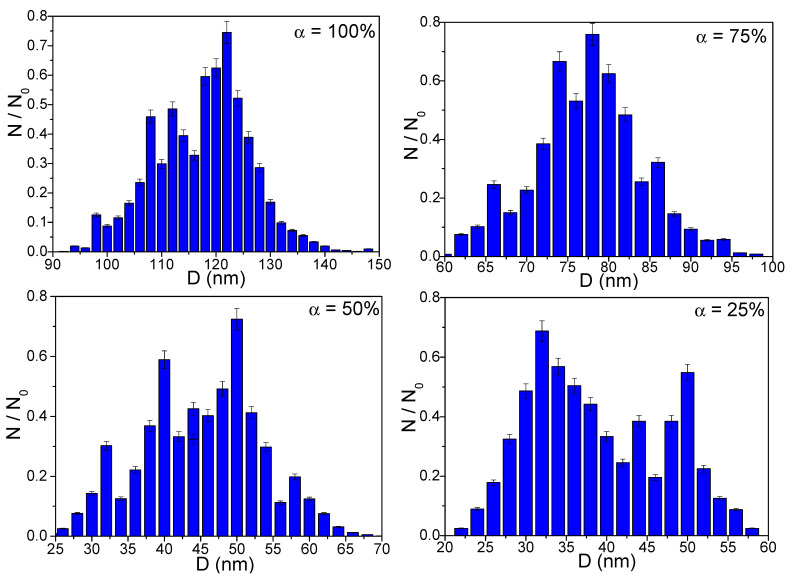
Particle size distributions for (Co_0.4_Zn_0.4_Ni_0.2_Fe_2_O_4_)_α_(SiO_2_) _100−α_ samples (α = 25–100%) annealed at 1200 °C.

The TEM image of the SiO_2_ matrix (α = 0%) consists of a dark area, without any possibility of identifying the matrix network, whereas those of samples α = 25–100% annealed at 500 and 800 °C are blurry, with low contrast, due to the small size poorly crystalline Co_0.4_Zn_0.4_Ni_0.2_Fe_2_O_4_ particles (~1 nm). For samples α = 25–100% annealed at 1200 °C (Figure 5), the higher ferrite content embedded in the SiO_2_ matrix results in large spherical particles. The increase in particle size from 34 to 122 nm (Table 1, Figure 6) with the ferrite content could be the outcome of different reaction kinetics, variation of the particle growth rate, or crystalline clusters formation [14,15,16,28]. The different particle arrangement could be due to the solid bodies formed by well-faceted grains, while the particle agglomeration could be a consequence of small particle size, inter-particle interactions, interfacial surface tensions, and strong intermolecular friction produced during the conversion of thermal energy into internal heat energy [14,15,16]. The porous surface formed by the gases generated during the thermal decomposition also favors the particle’s agglomeration [14,15,16].

The average crystallite sizes are consistent with the particle sizes determined from TEM, the differences being attributed to the interference in the diffraction patterns introduced by the amorphous SiO_2_ and large-size nanoparticles [12,13,14,15,16]. The particle size determined via TEM is generally larger than the crystallite size estimated by XRD, considering that a particle typically consists of several crystallites. The crystallite size can be calculated by analyzing the broadening of diffraction peaks without considering the effects of other factors that contribute to the diffraction peak width (i.e., instrumental contribution, temperature, microstrain, etc.) [29]. Moreover, even if they are few in number, the large nanoparticles significantly contribute to the diffraction patterns since they comprise a large fraction of atoms. The interference of the amorphous SiO_2_ with particle size lower than that of the embedded ferrite crystallites should also be considered [14,15,16].

**Figure 7:**
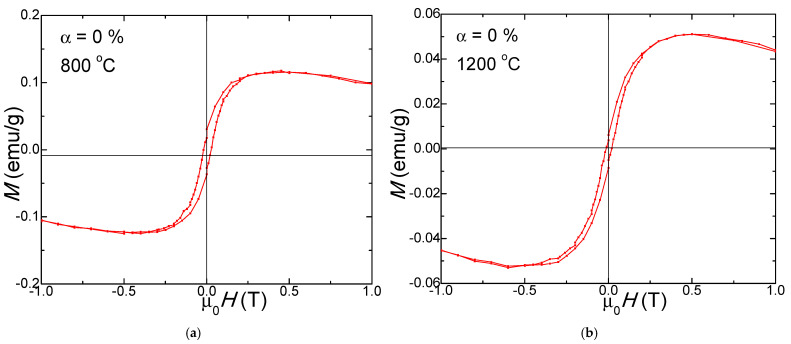
Magnetic hysteresis loops of SiO_2_ matrix (α = 0%) annealed at 800 °C (**a**) and 1200 °C (**b**).

**Figure 8:**
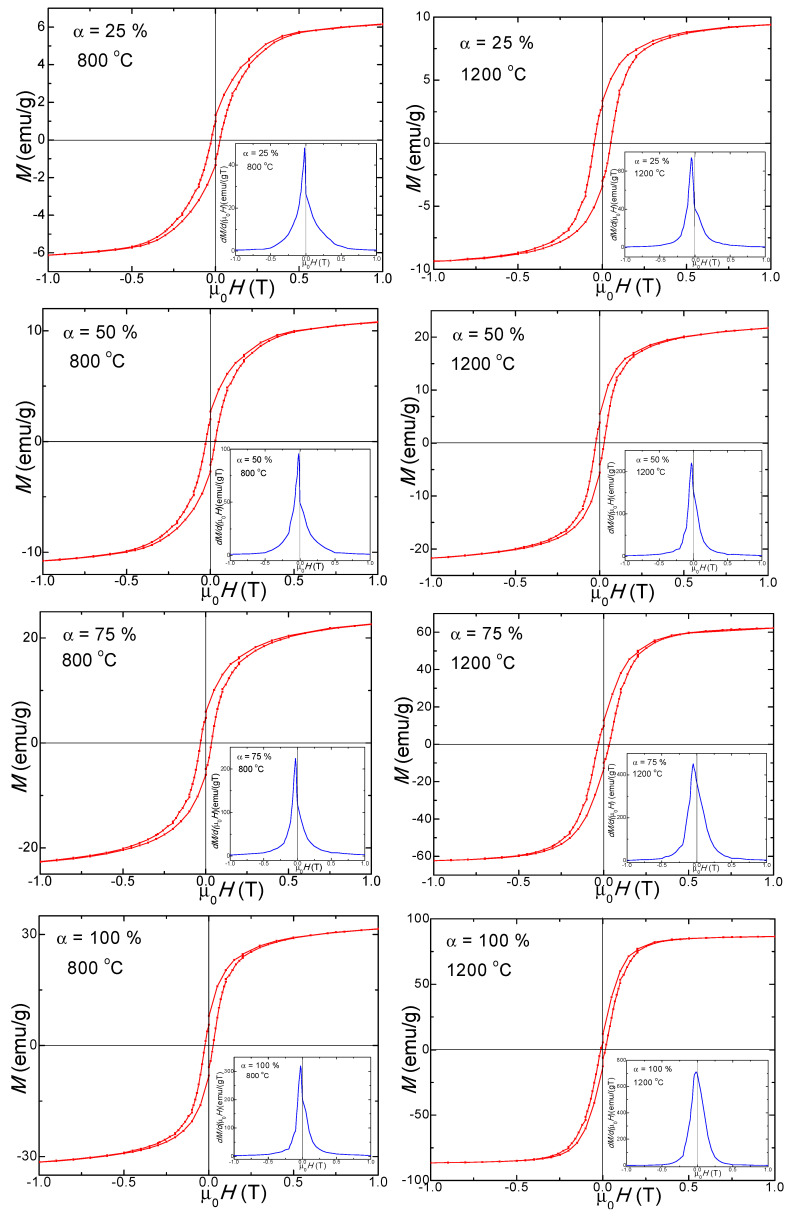
Magnetic hysteresis loops and magnetization derivative of (Co_0.4_Zn_0.4_Ni_0.2_Fe_2_O_4_)_α_(SiO_2_)_100−α_ samples (α = 25–100%) annealed at 800 and 1200 °C.

The SiO_2_ matrix (α = 0%) displays a diamagnetic behavior (Figure 7), while samples (α = 25–100%) show a typical ferromagnetic behavior (Figure 8) both at 800 and 1200 °C.

The samples show diamagnetic behavior in the presence of some accidental low concentration of ferromagnetic impurities, which likely come from the manipulation of the samples (from cutters, tweezers, spatula, etc.). The influence of these ferromagnetic impurities on the ferrites containing samples is negligible since the magnetization of the samples reaches 10 emu/g, while the ferromagnetic impurities can provide around 0.1 emu/g.

Generally, the magnetic properties of nano-ferrites are determined by the structure, crystal defects, porosity, particle size, and *K* [6]. The annealing temperature and SiO_2_ content are critical for producing single-phase nano-ferrites with enhanced magnetic properties. The *M_S_* increases with the Co_0.4_Zn_0.4_Ni_0.2_Fe_2_O_4_ content and in samples with the same composition with the annealing temperature [2,6,17]. The highest value of *M_S_* (90.1 emu/g) was obtained for non-embedded Co_0.4_Zn_0.4_Ni_0.2_Fe_2_O_4_ (α = 100%) annealed at 1200 °C. The increase of *M_S_* from 10.0 emu/g (α = 25%) to 90.1 emu/g (α =100%) with the ferrite content is a consequence of the non-magnetic SiO_2_ matrix that has a magnetic dilution effect [12,13,14,15].

The nanoparticle size is contingent on the annealing temperature, which controls the formation of assemblies of weakly interacting particles through reduced magnetostatic energy and weakly bonded interfaces [14,15,16,30]. According to Neel’s theory of ferrimagnetism, the magnetic properties of ferrites depend on the distribution of magnetic ions between the tetrahedral (A) and octahedral (B) sites [2,3]. The presence of impurity atoms or oxygen vacancies results in the break of the superexchange spin coupling between magnetic ions inducing a supplementary surface spin disorder [2]. Thus, magnetization is influenced by the crystalline structure, defects, and cationic distribution. The net magnetization of these spinel structures is given by the difference between the magnetic moments of tetrahedral (A) and octahedral (B) sites [5]. Magnetization is also affected by the migration of the magnetic ions between tetrahedral (A) and octahedral (B) sites and by the magnetic moment alignment from A and B sites [5]. When the Co^2+^ ions occupy the octahedral (B) sites, the magnetic moment (3μ_B_) will be higher in this position than in tetrahedral (A) sites which are occupied by Ni^2+^ and Zn^2+^ ions (2μ_B_) [3].

The higher ferrite content in samples annealed at 1200 °C results in an *M_R_* increase from 3.3 emu/g (α = 25%) to 13.7 emu/g (α = 100%). The remanence ratio *S* = *M_R_*/*M*_S_ indicates how square is the hysteresis loop. A theoretical value of this parameter *S* < 0.5 indicates samples consisting of an assembly of single domain, non-interacting particles [31,32,33]. As can be deduced from Table 2, this ratio is lower than 0.5 for all samples.

The *H_c_* increases from 260 Oe (α = 25%) to 320 Oe (α = 100%) with ferrite content embedded in the matrix in samples annealed at 800 °C and decreases from 485 Oe (α = 25%) to 165 Oe (α = 100%) in samples annealed at 1200 °C. The samples α = 50, 75, and 100% have small *H_c_* and high *M_S_* values due to the large particles with high magnetic coupling [31,32]. Generally, *H_c_* depends on crystallite sizes, magnetocrystalline anisotropy, domain walls and *M_S_* [3]. In the case of small single magnetic domain particles, they change the magnetization by spin rotation [34]. At high *H_c_* values, the thermal energy cannot induce the magnetization fluctuations for change of the magnetization [3]. Consequently, besides the particles’ shape and the density of disordered surface spins, *H_c_* has an important contribution to the magnetic order inside a monodomain particle. For large particles, the density of surface spins is low and results in higher magnetization [26].

The magnetocrystalline anisotropy constant (*K*) was calculated using Equation (3):

(3) $$K=\frac{\mu_{0}\cdot M_{S}\cdot H_{c}}{2}$$

where *M_S_* is the saturation magnetization, µ_0_ is vacuum permeability (μ_0_ = 1.256 × 10^−6^ N/A^2^), and *H_c_* is the coercivity field (T) [35].

The *K* values (Table 2) increase with the Co_0.4_Zn_0.4_Ni_0.2_Fe_2_O_4_ content embedded in the silica matrix, with the highest *K* observed for the non-embedded Co_0.4_Zn_0.4_Ni_0.2_Fe_2_O_4_ (α = 100%). The strain on the ferrite nanoparticle surface induced by the SiO_2_ matrix hinders the rotation of the magnetic moments from the particle-matrix interface [32]. As can be seen from Table 2, all the main magnetic parameters were strongly affected by the SiO_2_ embedding. *M_S_*, *M_R_*, and *K* depreciate substantially with the increase of SiO_2_ content due to the non-magnetic nature of the matrix. The *H_c_* value of the samples annealed at 1200 °C has a different trend; it is enhanced, suggesting the pinning of the magnetic moments from the surface of the particles, which is induced by the strain of the SiO_2_ layer.

The chemical composition, crystallographic structure, particle size, atomic packing density, and internal defects highly influence the ferrites’ magnetic properties [20]. Previous studies reported a decrease in the magnetocrystalline anisotropy of cubic ferrites when they are doped with Zn^2+^ ions [36]. Ni-Co ferrites have large *K* due to the Co^2+^ ions preference for the octahedral (B) sites [19].

The magnetization derivative curves (d*M*/d(µ_0_*H*)) vs. applied magnetic field are shown in the insets of Figure 8. The presence of a single peak indicates a single magnetic phase for samples α = 100%, considering that in a pure magnetic sample, the peak occurs at the nominal coercive field, suggesting crystalline samples with a single magnetic phase [14,16,31]. The sharp peaks indicate high magnetic purity, whereas the broad peaks suggest wide particle size distributions. The magnetization derivative for samples α = 25–75% annealed at 800 and 1200 °C indicate that two magnetic phases (from the triple Co–Zn–Ni ferrite) are magnetically coupled inside of the particle along their magnetic moments; the crystalline phases of Fe_2_SiO_4_ (a typical paramagnet at room temperature [34,37]), cristobalite and tridymite identified by XRD do not display magnetic properties, the hard magnetic phase being dominant (since it has a larger *H_c_*). If not instrumental, the peaks’ asymmetry reveals the presence of two magnetic phases inside the particles, one forming from a solid solution of two ferrites and another being the third ferrite.

In our previous studies on Co–Ni, Co–Zn and Zn–Ni ferrites, only a single magnetic phase was obtained [14,15,16]. For samples annealed at 1200 °C, the hysteresis loops are, generally, broader, and the (d*M*/d(µ_0_*H*)) vs. µ_0_*H* curves are narrower and sharper than in samples annealed at 800 °C. The peak heights and their horizontal shifts are related to the strength of the magnetic phases [14,26,30]. For samples annealed at 800 °C, the broader peaks suggest a large particle size distribution associated with a large *H_c_*.

The structure and the magnetic properties of (Co_0.4_Zn_0.4_Ni_0.2_Fe_2_O_4_)_α_(SiO_2_)_100−α_ are highly influenced by the SiO_2_ content and the annealing temperature. Our future studies intend to identify the metallic ion which does not couple with the other two metallic ions of the mixed ferrite leading to a second magnetic phase. Thus, by Co_0.4_Zn_0.4_Ni_0.2_Fe_2_O_4_ nanoparticles, the magnetic behavior can be easily tuned. Furthermore, combining the best magnetic properties and morphological configuration may be of interest for several technical applications.

## 4. Conclusions

The influence of SiO_2_ embedding on the structure, morphology, thermal, and magnetic properties of Co_0.4_Zn_0.4_Ni_0.2_Fe_2_O_4_ particles obtained by the sol-gel process was investigated. Fe, Co, Zn, and Ni malonates formed in two stages, as indicated on the DTA curve. At 1200 °C, the XRD and FT-IR results supported the formation of a single-phase spinel structure and SiO_2_ matrix. Highly crystalline single-phase ferrite starting from 300 °C for non-embedded Co_0.4_Zn_0.4_Ni_0.2_Fe_2_O_4_ (α = 100%) and an amorphous halo without any crystalline phases for SiO_2_ (α = 0%) sample were remarked. For samples α = 25–75%, the single crystalline phase Co–Ni–Zn ferrite at 500 °C was accompanied by Fe_2_SiO_4_ and quartz at 800 °C, while at 1200 °C the major cristobalite phase was accompanied by Co–Ni–Zn ferrite, tridymite, and quartz. The crystallite size increased with ferrite content in the SiO_2_ matrix, namely: 14.5–29.6 nm (500 °C), 26.3–52.4 nm (800 °C), and 33.3–118 nm (1200 °C), respectively. TEM images confirmed that the particles are in the nanometer range. The SSA gradually increased with the SiO_2_ content and decreased with the annealing temperature above 500 °C. The main magnetic parameters increased with the Co_0.4_Zn_0.4_Ni_0.2_Fe_2_O_4_ content: *M_S_* from 7.3 emu/g to 90.1 emu/g, *M_R_* from 1.2 to 13.7 emu/g, and *K* from 0.119∙10^−3^ to 0.934∙10^−3^ erg/cm^3^ (at 1200 °C). *H_c_* increased with Co_0.4_Zn_0.4_Ni_0.2_Fe_2_O_4_ content from 260 to 320 (at 800 °C) and decreased from 485 to 165 Oe (at 1200 °C). The *M_S_* and *M_R_* increased with the annealing temperature. As expected, the non-embedded Co_0.4_Zn_0.4_Ni_0.2_Fe_2_O_4_ (α = 100%) was ferromagnetic with high *M_S_*, while the SiO_2_ matrix (α = 0%) was diamagnetic with a small ferromagnetic fraction. Co_0.4_Zn_0.4_Ni_0.2_Fe_2_O_4_ non-embedded into the SiO_2_ matrix displays the behavior of a single magnetic phase, while the Co_0.4_Zn_0.4_Ni_0.2_Fe_2_O_4_ embedded in the SiO_2_ matrix shows two magnetic phases, the solid solution of two ferrites, and the third ferrite. Moreover, when the ferrite is embedded in the SiO_2_ matrix, the particle sizes decreased and the main magnetic parameters depreciated.

## Figures and Tables

**Table 1:** Particle size (D_TEM_), crystallites size (D_XRD_), crystallinity degree (DC), lattice constant (a), hopping length (L_A_ and L_B_), specific surface area (SSA), and Co/Zn/Ni/Fe molar ratio of (Co_0.4_Zn_0.4_Ni_0.2_Fe_2_O_4_)_α_(SiO_2_) _100−α_ samples.

Parameter	Temp. (°C)	α				
		0	25	50	75	100
D_TEM_ (nm)	1200	-	34 ± 2	50 ± 3	78 ± 4	120 ± 6
D_XRD_ (nm)	500	-	15 ± 1	17 ± 1	22 ± 1	30 ± 2
	800	-	26 ± 2	32 ± 2	42 ± 3	51 ± 3
	1200	-	33 ± 2	49 ± 3	75 ± 4	120 ± 5
DC (%)	500	51 ± 3	59 ± 3	65 ± 3	69 ± 4	76 ± 5
	800	61 ± 3	66 ± 3	68 ± 3	73 ± 4	79 ± 4
	1200	73 ± 4	81 ± 4	86 ± 4	91 ± 5	94 ± 5
a (Å)	500	-	8.40 ± 0.02	8.41 ± 0.02	8.43 ± 0.02	8.45 ± 0.02
	800	-	8.38 ± 0.02	8.39 ± 0.02	8.40 ± 0.02	8.43 ± 0.02
	1200	-	8.36 ± 0.02	8.37 ± 0.02	8.38 ± 0.02	8.40 ± 0.02
SSA (m^2^/g)	200	270 ± 14	220 ± 10	170 ± 9	24 ± 1	19 ± 1
	500	260 ± 14	270 ± 14	260 ± 14	120± 6	26 ± 2
	800	≤0.5	≤0.5	≤0.5	≤0.5	≤0.5
	1200	≤0.5	≤0.5	≤0.5	≤0.5	≤0.5
Co/Zn/Ni/Fe molar ratio	500	-	0.37/0.38/0.19/1.97	0.38/0.39/0.18/1.97	0.39/0.38/0.17/1.98	0.41/0.38/0.19/2.02
	800	-	0.31/0.29/0.18/2.20	0.32/0.30/0.17/2.33	0.39/0.38/0.18/2.03	0.41/0.38/0.19/2.02
	1200	-	0.39/0.38/0.18/1.99	0.39/0.41/0.19/1.99	0.39/0.40/0.19/2.01	0.41/0.41/0.20/2.00

**Table 2:** Saturation magnetization (*M_S_*), remanent magnetization (*M_R_*), coercivity (*H_c_*) and anisotropy constant (*K*) of (Co_0.4_Zn_0.4_Ni_0.2_Fe_2_O_4_)_α_(SiO_2_)_100−α_ samples annealed at 800 and 1200 °C.

Sample (Co_0.4_Zn_0.4_Ni_0.2_Fe_2_O_4_)_α_ (SiO_2_)_100−α_	Temperature (°C)	*M_s_* (emu/g)	*M_R_* (emu/g)	*S*	*H_c_* (Oe)	K·10^5^ (erg/cm^3^)
α = 25%	800	7 ± 0.4	1.2 ± 0.1	0.17 ± 0.02	260 ± 13	119 ± 6
	1200	10 ± 1	3.3 ± 0.2	0.33 ± 0.03	490 ± 25	305 ± 15
α = 50%	800	14 ± 1	2.6 ± 0.1	0.19 ± 0.02	270 ± 14	229 ± 11
	1200	29 ± 2	5.3 ± 0.3	0.18 ± 0.02	320 ± 16	577 ± 29
α = 75%	800	23 ± 1	5.7 ± 0.3	0.25 ± 0.03	300 ± 15	437 ± 22
	1200	62 ± 4	9.8 ± 0.5	0.16 ± 0.02	220 ± 11	857 ± 43
α = 100%	800	38 ± 2	8.7 ± 0.4	0.23 ± 0.02	320 ± 16	754 ± 38
	1200	90 ± 6	14 ± 1	0.15 ± 0.02	170 ± 10	934 ± 47

## Data Availability

Data are available on request from the corresponding author.
